# Extraction of heavy metal complexes from a biofilm colony for biomonitoring the pollution

**DOI:** 10.3906/kim-1912-38

**Published:** 2020-06-01

**Authors:** Sedat SÜRDEM, HacıMehmet DOĞAN

**Affiliations:** 1 National Boron Research Institute, Ankara Turkey; 2 Department of Chemistry, Faculty of Science, Hacettepe University, Ankara Turkey

**Keywords:** Extraction, heavy metal, biofilm, biomonitoring, pollution

## Abstract

An extraction method was tested for biomonitoring the biofilm samples containing heavy metals. The fractionation of metal complexes was performed via C-18-HPLC-ICP-MS and MALDI-MS, respectively. The extraction power of some reagents was determined for the heavy metal extraction from biofilm samples collected in Erdemli coast in the Mediterranean Sea. The ammonium acetate solution giving the highest extraction results was found as a suitable extraction reagent. The concentration and pH of the ammonium acetate solution were optimized and found as 1 M and 5, respectively. The chromatograms of metal complexes with the C-18-HPLC-ICP-MS system were taken to determine the effect of the pH of the metal complexes. After performing the extraction, metal bounded biomolecules were characterized by MALDI-MS for the fractions in the C18-HPLC system. It was seen that ammonium acetate extraction (1M, pH 5) might be used in biomonitoring studies due to relatively simple procedure, short analysis period, and low cost. The evaluation of the applicability of the method in biomonitoring studies might be supported by further studies with biofilms having similar characteristics.

## 1. Introduction

Heavy metal pollution has been growing, and its release into the environment on a large scale has become an alarming issue. The mean contaminant concentration of seawater cannot be regarded as a reliable measure of related location [1]. Trace metal concentrations were determined in water, biofilms, and sediment matrices of the Bílina River in the Czech Republic [2]. In a recent study, heavy metal levels were investigated by digesting surface sediments on the Mediterranean coast of Morocco [3]. Sediment analysis provides total contaminant load instead of direct ecotoxicological relevance. Concentrations of pollutants may not be correlated with data from sediments in the overlying water column [4]. The relative growth, metal accumulation, and tolerance of different plant parts were examined [5]. The relation between the elemental composition of biofilms (algal biomass) and the water column was tested in a study [6]. Variations in physical, chemical, and biological properties are directly and sensitively observed with a microalgal response. They are excellent indicators sensitive to several environmental variables, including heavy metals [7,8]. Microalgae are capable of accumulating a significant amount of metals [9], and they have some other important characteristics such as rapid growth, wide habit, etc., to be used as bioindicators [10].

Biofilms are composed of algae, bacteria, etc., their extracellular polymeric substances (EPS) and nonliving materials [11–13]. Metals are included almost in all parts of the biofilm samples. Especially, EPS of algae in mature biofilms [14] is very effective in metal complexation, so reduces the bioavailability of metals and their toxicity to algal cells.

The capacity of algae both in living and nonliving form on a metal-binding is very high and effective. The first step in metal uptake starts with the adsorption of ions or complexes on a cell wall. Active transport of metal (either essential or toxic) into phytoplankton cells is realized with a chelator. Responsibility for algal survival in metal-polluted environments has been described with several mechanisms; increases in the extracellular metal chelators production [15–17], metal immobilization with binding at the cell surface [18,19] or tolerance in internal mechanisms of storage and detoxification [20].

MT (metallothioneins) induction was carried out to a single-metal exposure in most experiments [21–25]. However, assessments of MTs should include metal mixtures if used as a biomarker. In that case, it might reflect the actual hazard of contamination when compared to a single toxicant. Studies with MTs response to metal mixtures are scarce. Lecoeur et al. [26], studied the influence of metal mixtures, as Cu+Cd, on MT response, and compared the results with single-metal exposure for the bivalve D. polymorpha.

All the higher and lower plants were analyzed in terms of the induction of PCs (phytochelatins) with the presence of intracellular heavy metals [27–31]. Recently, the online coupling of HPLC with ICP-MS has been tried for characterizations of heavy metal-binding peptides. Due to its high resolution, the most commonly used HPLC technique is the reversed-phase, as far as the PC separation is concerned [32,33]. Krauss et al. [34] extracted and measured the thiol-containing peptides according to the lines of Grill et al. [35], and reversedphase Cl8-HPLC-ICP-MS was used for the detection. Speciation of 4 metals has been studied for sediments in Egypt using sequential extraction with a 4-step procedure [36].

In this study, the contents of 8 different metals in biofilms were determined in the extracted form after obtaining a suitable extraction reagent and optimizing its concentration and pH of its solution. Moreover, the usability of the biofilm for the extraction of heavy metals from biofilm samples was also checked. Fractionation and determination of metal complexes without destroying the structure in the biofilms were also studied with C-18-HPLC separation and Matrix-assisted laser desorption/ionization-mass spectrometry (MALDI-MS) characterization.

## 2. Materials and methods

### 2.1. Preparation of biofilm samples

**Figure 1:**
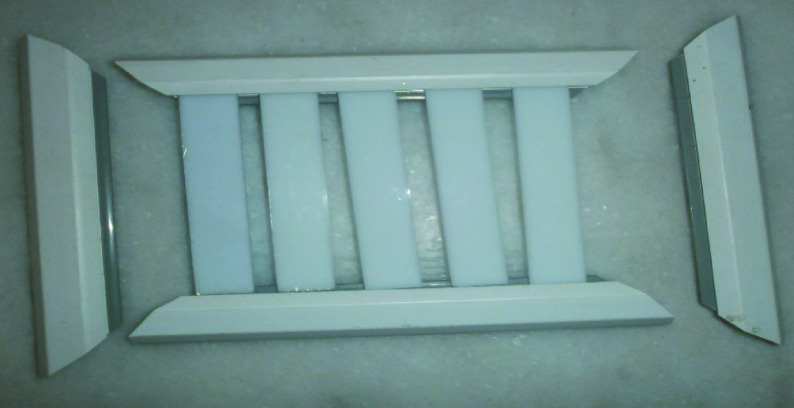
PVC frames with plexiglass slides.

Plexiglass slides (artificial substrate) were used to accumulate biofilm. They were supported by polyvinyl chloride (PVC) holder with plastic clips. PVC was prepared in a rectangular frame (Figure 1). For each station, substrates within PVC frame support were dipped in water with a depth of 40–50 cm for 60 days. Then, plates were taken all at once.

### 2.2. Digestion of biofilm samples

Dry algal sample (0.10 g) was accurately weighed and decomposed with a mixture of 65 % HNO_3_ (Merck), 35 % H_2_O_2_ (Merck), and 37 % HCl (Merck) with the volume percent ratios of 60/20/20, respectively. Digest was quantitatively transferred into volumetric flasks after cooling. It was completed to a final volume of 25 mL with deionized water. After decomposition, the supernatant was centrifuged for 15 min. It was waited to a constant room temperature and then again diluted 10 times with DW when measured in the instrument. Blank and standard solutions of the metals were also prepared in the same conditions. Signal of blank, standards, certified standard material and samples were observed and the total heavy metal contents of sample were determined in ICP-MS instrument. The instrument was calibrated using multi-element standards solutions prepared by mixing HNO_3_, HCl, and H_2_O_2_ and diluting ICP-MS stock solutions of individual elements with the proper ratio of sample content. Aliquot of digest (5 mL) was spiked with internal standard (indium) with a final concentration of 5 μg/L and completed to 10 mL with deionized water. Standard solutions of in 0.01, 0.1, 1.00, 10.00, and 100.00 ng/mLconcentrations were prepared from their stock solutions. Indium was also added to these calibration standard solutions as an internal standard to monitor and compensate for the possible instrumental drift. Various isotopes were selected and used for the determination of the related elements.

### 2.3. Selection the extraction reagent and optimization of parameters

Three kinds of reagents (ammonium acetate, ammonium nitrate, potassium sulphate) were selected for the desorption of metals from algal biofilm samples. The concentration of each reagent was 0.1 M in 10 mL. For each treatment, 100 mg of algal samples (weighing is performed after stripping algae from the plexiglass slide surfaces) were added to each solution. Mixtures were kept at 4 °C in a final volume of 10 mL during the extraction step. Samples were put into a shaker, and the tube was shaken for 30 min with an ultrasonic shaker and centrifuged at 5000 rpm for 15 min at 4 °C. Afterward, the supernatants were filtrated using a 10 KDa ultrafiltration membrane (Filter Code: YM10 Dia: 63.5 mm) and Millipore Stirred ultrafiltration cell (8400 Model) to obtain a clear solution. The metal contents of the filtered solutions were determined in ICP-MS after the instrument was calibrated using multi-element standards solutions prepared in different concentrations of ammonium acetate solutions. Aliquots of extracts (8 mL) were spiked with internal standard (indium) and completed to 10 mL with deionized water.

### 2.4. Separation of extracted metals with HPLC

The column chosen for the separation of the metal complexes was ion-pairing C18 (Dionex C18); heptafluorobutyric acid (HFBA) was selected as the ion-pairing reagent. In the C18-HPLC-ICP-MS system, 10.0% of CH_3_OH in 0.12% HFBA was used as a mobile phase at natural pH (pH = 6.0). The flow rate of mobile phase was adjusted to 1.2 mL/min.

### 2.5. Characterization of separated metal complexes in MALDI-MS

MALDI matrix sinapinic acid (SA, 3,5-dimethoxy-4-hydroxycinnamic acid) was prepared in an acetonitrile:water: trifluoroacetic acid mixture (1:1:0.001, v/v) at a concentration of 10 mg/mL. MALDI samples were prepared by mixing HPLC fractions with the matrix solution (1:10 v/v) in a 1.5 mL Eppendorf microtube. Finally, 1.0 μL of this mixture was deposited on the sample plate, dried at room temperature (RT), and then analyzed for heavy metal complexes. Mass spectra were acquired on a Voyager-DE PRO MALDI-TOF mass spectrometer (Applied Biosystems, USA) equipped with a nitrogen UV-Laser operating at 337 nm. Spectra were recorded in linear mode with an average of 100 shots.

## 3. Results and discussion

### 3.1. Extraction of heavy metals from biofilm colony

Instead of quantifying metals simply as adsorbed or absorbed in algal cells, we preferred to determine the metals in both forms at the cell surface and within the cell. The extraction procedure for total metal concentration was given with the optimized parameters in this study.

A few chemicals to desorb the metals bound to molecules’ physical or chemical either in the ion-pair or complex form were examined. Optimizations of concentration and the pH of the selected desorption reagent were also performed in the experiments.

Three kinds of reagents were selected for the desorption of metals from algal biofilm samples. Ammonium acetate, ammonium nitrate, and potassium sulphate were the reagents, pH of which are near neutral and are thought as unaffected while the treatment.

### 3.2. Selection of extraction agent for metal complexes

The suitability of the selected chemicals was examined to be used as an extraction agent for the metal complexes. The concentrations of the reagents were prepared as 0.1 M in 10 mL for each metal complex. The extraction power of 3 reagents is selected as seen in Table 1. It was concluded that the ammonium acetate solution was the extractant, which provided the highest extraction recovery among the solutions studied.

**Table 1:** Amounts of metals extracted with different reagent solutions from the biofilm samples (for standard deviations: n = 3. Extracted samples were measured without further dilution except for solid-liquid extraction procedure).

Metal (mg/kg)	Ammonium acetate	Ammonium nitrate	Potassium sulphate
Cr	1.52 ±0.10	1.36 ±0.06	0.25 ±0.02
Fe	19.80 ±1.40	13.40 ±0.40	9.91 ±0.32
Mn	52.19 ±2.30	44.30 ±1.80	15.90 ±1.00
Ni	2.91 ±0.23	2.15 ±0.24	0.89 ±0.09
Cu	2.11 ±0.09	1.29 ±0.07	0.64 ±0.07
Zn	20.50 ±0.40	18.90 ±0.80	6.56 ±0.43
Cd	0.05 ±0.01	0.02 ±0.01	0.02 ±0.01
Pb	0.67 ±0.04	0.61 ±0.12	0.25 ±0.03

The most remarkable difference has appeared in the iron, cadmium, and copper extractions. It was about 50, 80, and 150% higher with the ammonium acetate reagent than with ammonium nitrate with the significant uncertainty in Cd concentration, respectively. The concentrations of all metals with the potassium sulphate reagent had the lowest values in the extraction. We observed that the extraction recoveries were too low to evaluate the metal complexes in the instruments worked. The reason for the low recoveries was probably due to the low concentration values of the extraction reagents and high pH. Determination of the total content of each metal in the extraction procedure was not possible since the extraction recoveries of metals were much lower than 100%. Nevertheless, the extraction of metals without destroying their complex structures gives an idea about both absorbed and adsorbed metals, separately. Additionally, it was possible to see each metal of the original structure in biofilm.

### 3.3. Optimization of the concentration of ammonium acetate solution

In this part of the extraction study, the concentration of the selected extraction solution was optimized to obtain the best extraction efficiency. Better extraction results based on ammonium acetate concentration were investigated to reach a reasonable extraction value. The concentration range was limited to appreciable values, which were 0.1, 0.5, and 1.0 M, while the pHs of the solutions were kept constant near to the neutral value of pH 7.0. Values below 0.1 M and above 1.0 M were not studied since the lower values approach to the behaviour of water, and higher values may block the instrument.

A suitable condition value for ammonium acetate concentration was evaluated to increase the extraction of metals. In the previous part, the concentration of extraction reagent (ammonium acetate) was prepared as 0.1 M and it was seen as too low for the extraction of the metals from the results. Two other concentration values were also tried to increase the extraction efficiencies of the metals. Results of 3 values such as 0.1, 0.5, and 1.0 M are given in Table 2.

**Table 2:** Amounts of metals extracted with ammonium acetate solutions (pH = 7) with different concentrations from the biofilm samples (for standard deviations: n = 3. Extracted samples were measured without further dilution except for solid-liquid extraction procedure).

Metal (mg/kg)	The concentration of ammonium acetate			
	0.1 M	0.5 M	1.0 M
Cr	1.52 ±0.10	2.32 ±0.14	5.16 ±0.21
Fe	19.81 ±1.35	27.59 ±0.43	37.30 ±2.14
Mn	52.19 ±2.30	80.49 ±2.02	118.73 ±6.37
Ni	2.93 ±0.16	4.12 ±0.27	5.48 ±0.28
Cu	2.11 ±0.12	3.56 ±0.28	5.39 ±0.27
Zn	20.49 ±1.08	55.91 ±3.15	75.45 ±4.89
Cd	0.05 ±0.02	0.21 ±0.03	0.54 ±0.05
Pb	0.67 ±0.04	2.57 ±0.14	3.04 ±0.12

It showed that the concentration of ammonium acetate solution had a serious effect on the extraction of metals. The higher concentration of ammonium acetate was recorded as having the highest recoveries. Nevertheless, concentration values above 1.0 mol.L^-1^ for ammonium acetate were not tried since high levels of salt concentrations may plug the way to plasma and stop it in off position. Therefore, ammonium acetate solution in 1.0 mol.L^-1^ concentration was decided to be used for the further extraction studies.

### 3.4. Optimization of pH of ammonium acetate solution

Ammonium acetate solutions in 1.0 M concentration were prepared at different pH values to find out the effect of the pH on the extraction of metals as seen in Table 3. When compared with the extraction results of ammonium acetate solutions prepared at different pH values, it was obvious that there was a reverse relation between the pH and the extraction values as expected. Although each change in pH would not result in a change in the extraction values for some of the conditions, a continuous increase in the extraction of the metals was detected for many of the gradual decreases in pH.

**Table 3:** Amounts of metals extracted from biofilms with ammonium acetate solution (1M) with different pH values (for standard deviations: n = 3. Extracted samples were measured without further dilution except for solid-liquid extraction procedure).

Metal (mg/kg)	The pH of ammonium acetate solution		
	7.0	6.0	5.0
Cr	6.81 ±0.29	7.09 ±0.11	8.50 ±0.21
Fe	51.00 ±2.40	72.7 ±3.00	79.20 ±2.10
Mn	134 ±6	196 ±13	183 ±8
Ni	5.46 ±0.19	5.52 ±0.38	6.21 ±0.28
Cu	7.24 ±0.67	6.92 ±0.07	7.08 ±0.27
Zn	84.00 ±4.20	98.4 ±5.90	127 ±5
Cd	0.73 ±0.04	0.76 ±0.02	0.81 ±0.08
Pb	3.05 ±0.16	3.11 ±0.32	3.71 ±0.12

The Cell wall matrix of algae contains complex hetero-polysaccharides, which offer carboxyl and sulphate groups. Since deprotonated free carboxyl or sulphate groups were negatively charged, they would electrostatically attract any cation [37]. The magnitude of the overall negative charge increases as the pH increases. Therefore, more sites were deprotonated for the adsorption of metals. Due to these properties of algal cell walls, algal biomass may accumulate metals in the cationic form in the aqueous media in the sea. The accumulated metals in the algal cell walls in the sea may be desorbed when the biosamples were contacted with a solution having a high hydronium ion or salt concentration after they were dried and homogenized in the laboratory.

Considering the effect of the pH and concentration of ammonium acetate reagent together, a continuous increase in the extraction of metal complexes becomes not surprising due to the high concentrations of salt and hydronium ion. As expected, the ammonium acetate solution to desorb the metal complexes which were not only adsorbed in the cell walls of the algal biosamples extracellularly but also absorbed in the cells intracellularly as the phytochelatin complexes.

When the pH was changed from 7.0 to 6.0, the desorption values of Cr, Cu, and Cd were almost constant. An interesting result was seen in the Mn-extraction. It was lower in the pH 5.0 than 6.0, unexceptionally. Lower values of pH of the solution were recorded as having the highest recoveries. Also, pH values below 5.0 were not analysed since decreasing pH may create the deformation of the metal complexes. Therefore, ammonium acetate solutions with 1 M concentration were decided to be used at a pH of 5.0 for further extraction studies. To determine whether metal complexes deform or not, extracted metals and their complexes should be separated and characterized.

### 3.5. Extraction efficiency

The total digested content of selected 8-heavy metals was also determined to compare the results with the extracted metal contents of biofilm samples as seen in Table 4. The sequence of the contents of 8-heavy metals in biofilm has appeared as Fe>Mn>Zn>Cu>Cr>Ni>Pb>Cd.

**Table 4:** Comparison of the ammonium acetate (1 M, pH 5.0) extracted metals with the acid digested ones from the biofilm samples (for standard deviations: n = 3. Digested samples were measured after 100-fold dilution. Extracted samples were measured without further dilution except for solid-liquid extraction procedure).

Metal	Extracted	Digested	Extracted/digested (%)
Cr	8.5 ±0.2	18.5 ±1.1	45.9
Fe	79.2 ±2.1	1768 ±91	4.5
Mn	183 ±8	495 ±11	37.0
Ni	6.2 ±0.3	10.3 ±0.3	60.1
Cu	7.1 ±0.3	19.5 ±1.3	36.3
Zn	127±5	241. ±21	52.8
Cd	0.8 ±0.1	4.6 ±0.2	17.7
Pb	3.7 ±0.1	8.9 ±0.5	41.4

The extraction values of the metals were given in the extraction part of the study after the parameters affecting the extraction recovery were optimized. As the concentrations of the metals were thought as taken totally after dissolved in the digestion, the percent extracted values of the metals would be given in terms of total values. Percent extraction values were calculated to form maximum recovery values per total digested values. Extraction efficiencies range from about 5% to 60% for different metals as seen in Table 4. The highest recovery value of 60% belonged to Ni. The lowest recovery was found for Fe concentration with 5%, and the nearest one was Cd recovery with about 18%. The percent recovery values of Cu and Mn were very close to each other at around 36%. Pb, Cr, and Zn recovery values were somehow close to each other, and they were 41%, 46%, and 53%, respectively. As a summary, the sequence for the recovery values was in the order of Ni>Zn>Cr>Pb>Mn>Cu>Cd>Fe.

There would be a few reasons to have different recovery values for each metal for the same sample. Indeed, the amount of metal sorbed in total components of biofilms may probably vary with metal and their concentrations distributed as EPS, cell wall, and intracellular metal complexes in biofilm. Percent recovery value for each metal depends on its chemical and/or physical form in which the bound structure is mostly affected with the extraction reagent. Although our suggestion for extraction values instead of digestion ones may also be used in biomonitoring studies due to relatively simple procedure, short analysis period, and low cost, more studies with biofilms having similar characteristics should be applied for the evaluation of the applicability of extraction procedure in biomonitoring studies.

### 3.6. Separation of extracted heavy metal complexes

After the determination of extraction agent and optimization of its concentration, pH value was not defined for extraction since there was a continuous increase in the desorbed metal content of the sample, while the pH of the agent was decreased step by step. Not knowing the effect of pH of ammonium acetate in the structure of desorbed metal complexes, whether it decomposes the structure or not, all the supernatants of solutions at different pH values were observed in HPLC-ICP-MS system.

The effect of pH on the extractions of metals was discussed in the previous sections, and there was a gradual increase in the extraction of most metals with decreasing the pH of the ammonium acetate solution. The chromatograms of metal complexes from 3 solutions (pH: 7.0, 6.0, 5.0) in the C-18-HPLC-ICP-MS online system were taken and compared in this part of the work to determine the effect of pH on the metal complexes.

**Figure 2:**
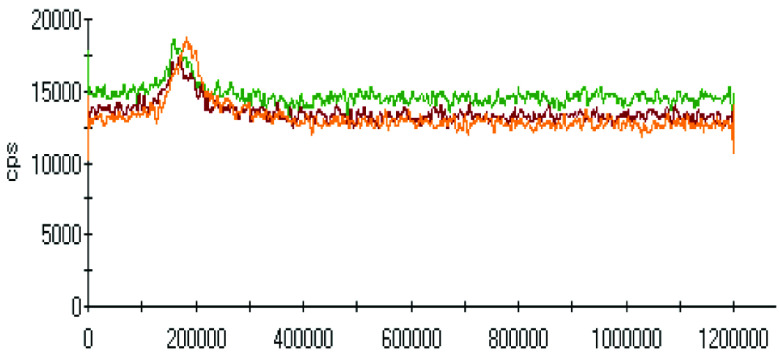
Chromatograms of Cr extracted at 3 different pH values.

**Figure 3:**
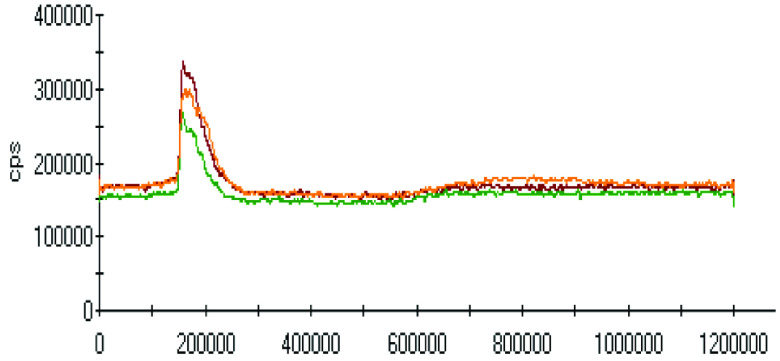
Chromatograms of Fe extracted at 3 different pH values.

**Figure 4:**
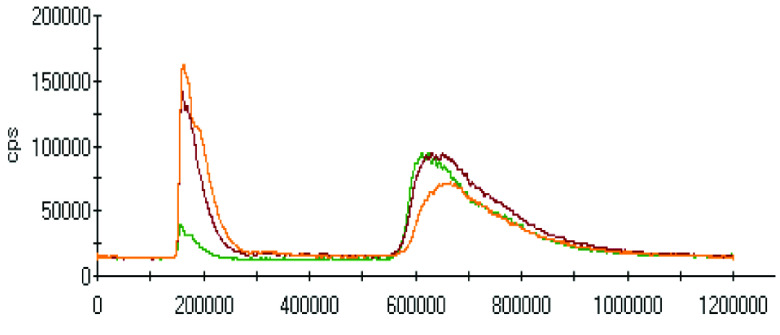
Chromatograms of Mn extracted at 3 different pH values.

**Figure 5:**
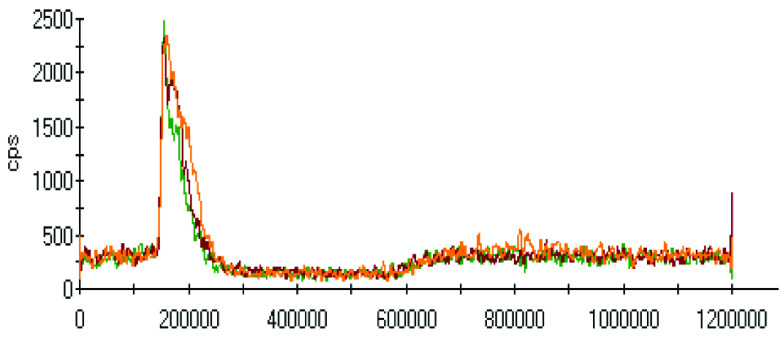
Chromatograms of Ni extracted at 3 different pH values.

**Figure 6:**
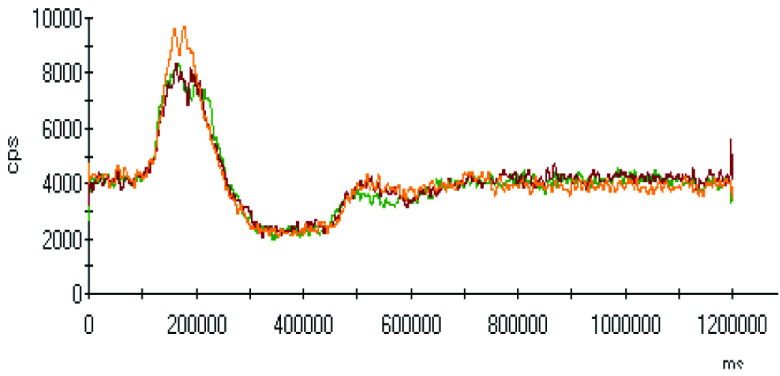
Chromatograms of Cu extracted at 3 different pH values.

As chromatograms of metals at different pH values were compared (Figures 2–9), some of them show no change in peak areas, whereas others have different chromatograms like variations in the extraction results. While the peaks in the chromatograms of Cu and Cd were observed as not much changed, those of others were recorded as more or less changed. One peak was observed for Cr, Fe, Ni, Cu, Cd, and Pb metals separately, whereas Mn and Zn have 2 peaks in their chromatograms. Whether there are unresolved peaks or not, we wanted to observe the peak areas whether or not they match the extraction results at the same pH values. Although the areas of the extracted metals in HPLC-ICPMS do not match the results of extracted metals without HPLC, tendencies of results are parallel to each other. The difference in nebulization efficiencies with different reagents (water in extraction and methanol-water-HFBA mixture for HPLC) is the main reason for reaching different results. Since organic solvents mostly have high nebulization efficiencies due to the low surface tension, metal peak areas in an organic solvent are recorded as higher than those in water.

**Figure 77:**
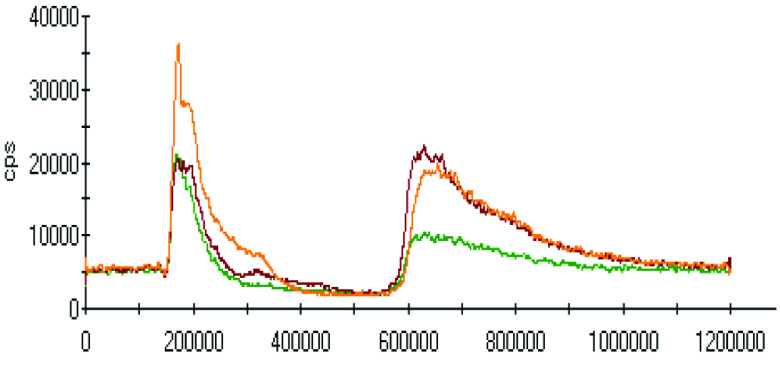
Chromatograms of Zn extracted at 3 different pH values.

**Figure 8:**
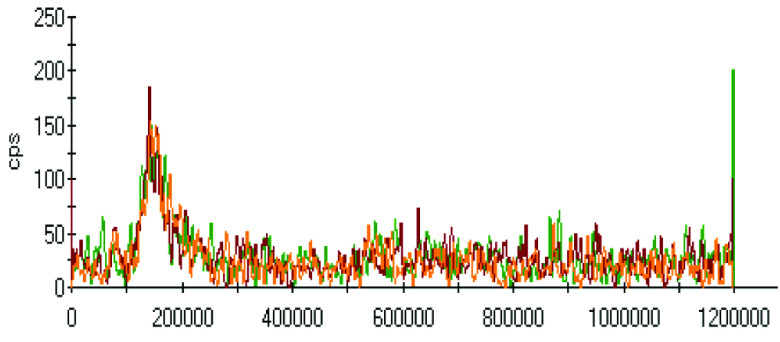
Chromatograms of Cd extracted at 3 different pH values.

**Figure 9:**
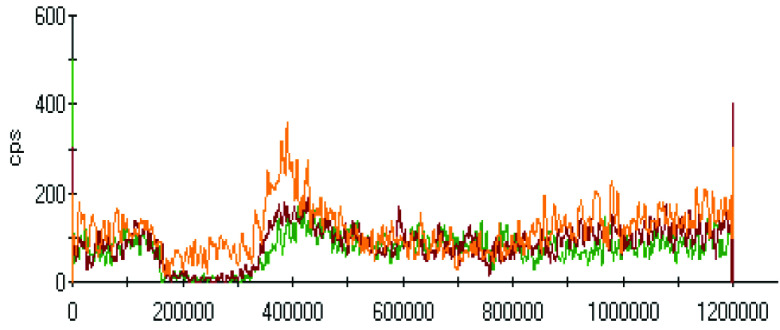
Chromatograms of Pb extracted at 3 different pH values.

Orange, red and green colors were related to pH values of 5.0, 6.0, and 7.0, respectively in all chromatograms.

As the chromatograms of metals were observed, fascinating results were recorded. A peak was seen at a retention time of 400 s in which no metal had a certain signal except lead chromatogram of extraction sample, which is hardly detected due to the low signal to noise ratio to evaluate. Therefore, selective isolation, determination, and characterization of lead complexes may be possible if the signals are enhanced with increasing the sample amount and/or decreasing pH.

Observing the peaks near 700 s for Mn and Zn chromatograms is another interesting result due to the signals just belonging to these metals. Although a considerably high increase was observed from pH 7.0 to 6.0 in the first peak, it seems similar in the second one in the Mn chromatogram. Another attractive result was marked in the change from pH 6.0 to 5.0. While they look similar in the first peak, the peak was lower in pH 5.0 in the second retention time for Mn. Peaks are similar at pH 6.0 and pH 7.0 for chromatograms of Zn; however, a specific increase was observed at pH 5.0 at a retention time of about 200 s. The signals were recorded as similar at pH 5.0 and pH 6.0 chromatograms of Zn, however, a certain decrease was observed at 700 s signal of pH 5.0.

Even though a little peak with a big noise is seen below 200 s for Cd, it is very obvious that Cd levels in the biofilm and extracted solution are too low to evaluate with the conditions performed. The peak was though not meaningful with a signal to noise ratio of about 3. It was clear that observing the change in signals in different chromatograms was unsuitable. Moreover, it seems unchanging while the conditions change.

### 3.7. Characterization of separated heavy metal complexes by MALDI-MS

Selection of the proper matrix is important in MALDI-MS, because different matrices allow analysis of varying types of biomolecules [38]. SA is routinely used in profiling experiments of biomolecules, although other matrices (CHCA, and ferulic acid) can be used. SA, CHCA, and ferulic acid have been shown to be useful for the detection of proteins [38,39]. SA matrix was selected for the characterization of the metal bound MTIII metallothioneins having molecular weights ranging from 2 to 10 kDa and recognized as peptides induced most strongly by metals in the algae. Mixures of MALDI matrix SA (in an acetonitrile:water:trifluoroacetic acid mixture) and HPLC fractions (1:10 v/v) were prepared in 1.5 mL Eppendorf microtubes. 1.0 μL of each mixture was deposited on the sample plate, dried at room temperature (RT), and then analyzed for heavy metal metallothionein complexes.

Ammonium acetate solution with 1.0 M concentration at pH 5.0 used in the extraction step was used to characterize the metal bounded biomolecule forms by MALDI-MS after the fractions were taken from the C18-HPLC system. The chromatograms can be investigated as dividing into 3 fractions since peaks appeared in 3-regions. The first fraction was taken from the start to 300 s and the second one started from 300 to 500 s. The last fraction was collected from 500 up to 1000 s. These 3 fractions were analyzed to characterize the metal complexes. Methanol, HFBA, and water mixture (5%, 10mM, 95%, respectively) were used in the HPLC separation step.

**Figure 10:**
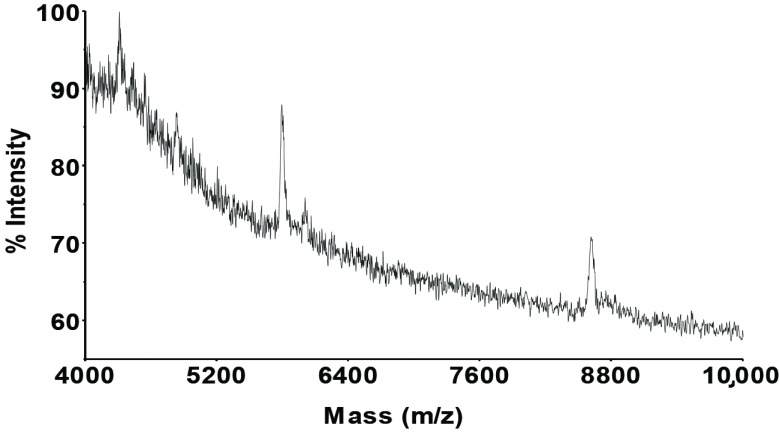
MALDI-MS spectrum of the first fraction from the chromatograms of extracted metals from the biofilm sample.

Almost all the metals have signals in the HPLC-ICP-MS system in the first fraction, and so the signals in the MALDI- MS system may be related to these metals (see Figure 10).

**Figure 11:**
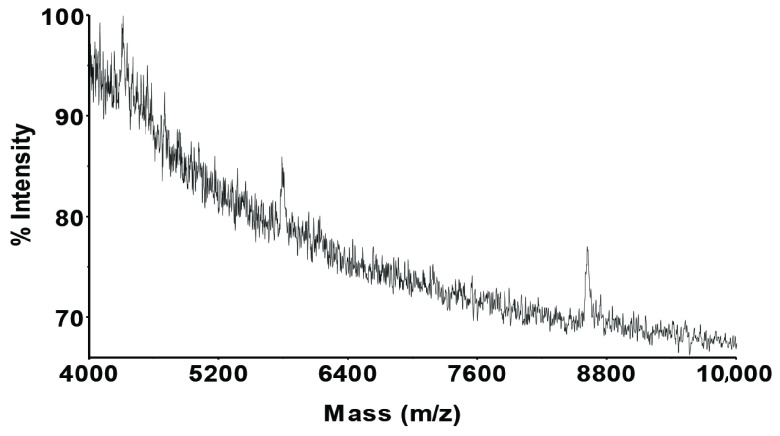
MALDI-MS spectrum of the third fraction from the chromatograms of extracted metals from the biofilm sample.

The second fraction was very interesting since the only peak appeared in the lead chromatogram. However, certain signal(s) was/were not observed in the MALDI-MS instrument due to very low concentrations of lead in the fraction. Only Zn and Mn peaks were recorded in the last fraction of the chromatograms. Therefore, the peaks in the MALDI-MS may be related to these metal and their complexes (see Figure 11).

In each fraction (fraction I and fraction III), MALDI-MS spectra showed an almost similar spectrum, which had some mass shifts on the similar biomolecule-metal complexes that appeared at around 4296, 5779, and 8611 Da masses with + 5 Da mass deviations onto the first 2 peaks. These mass deviations might be the different metal complexes of the same biomolecule complexes, such as from manganese to zinc.

## 4. Conclusions

Percent recovery value for each metal depends on its chemical and/or physical form in which the bound structure is mostly affected with the extraction reagent. It was found that extraction values could be used in biomonitoring studies due to their advantages. Nevertheless, more studies with biofilms should be applied for the evaluation of the applicability of the extraction procedure in biomonitoring studies.

Metal chromatograms of the C-18-HPLC-ICP-MS system can be used for each metal determination. It is very interesting that no metal had a certain signal except lead chromatogram of the extraction sample. This relates to the separation of Pb complex from other metal complexes. Therefore, selective isolation, determination, and characterization of Pb complexes may be possible. Observing the peaks near 700 s for Mn and Zn chromatograms is another interesting result due to the signals just belonging to these metals. Even though a little peak with a big noise is seen below 200 s for Cd, it is very clear that Cd levels in the biofilm and extracted solution are too low to evaluate with the conditions performed.

The second fraction form HPLC was very interesting since the only lead peak appeared in the chromatogram. However, the signal was not observed in MALDI-MS due to very low concentrations of lead in the fraction. However, it can be used in the work where the Pb concentration is higher than this work. Only Mn and Zn peaks were recorded in the last fraction of the chromatograms. Therefore, the peaks in the MALDI-MS may be related to these metals and their complexes. When the metal-biomolecule complex peaks were examined in detail, it could be noticed that each complex peak did not occur. It also had some isotopic mass distribution, such as metal isotopic peak distribution. This showed that the type of metal ion in each MALDI-MS peak could be identified when the high resolved mass spectra were obtained.
